# Umbilical vein injection of misoprostol versus normal saline for the treatment of retained placenta: intrapartum placebo-controlled trial

**DOI:** 10.1186/1471-2393-14-37

**Published:** 2014-01-21

**Authors:** Sheelan S Rajab, Shahla K Alalaf

**Affiliations:** 1 Department of Obstetrics and Gynaecology, Shaheed Dr.Khalid General Hospital, Erbil City, Iraq; 2 Department of Obstetrics and Gynaecology, College of Medicine, Hawler Medical University, Erbil City, Iraq

**Keywords:** Intraumbilical misoprostol, Retained placenta, Active management of third stage of labour

## Abstract

**Background:**

The third stage of labour may be complicated by retained placenta, which should be managed promptly because it may cause severe bleeding and infection, with a potentially fatal outcome. This study evaluated the effectiveness of umbilical vein injection of misoprostol for the treatment of retained placenta in a hospital setting.

**Methods:**

This hospital-based placebo-controlled trial was conducted at the Maternity Teaching Hospital, Erbil City, Kurdistan region, Northern Iraq from April 2011 to February 2012. The inclusion criteria were: gestational age of at least 28 weeks, vaginal delivery, and failure of the placenta to separate within 30 minutes after delivery of the infant despite active management of the third stage of labour. Forty-six women with retained placentas were eligible for inclusion. After informed consent was obtained, the women were alternately allocated to receive umbilical vein injection of either 800 mcg misoprostol dissolved in 20 mL of normal saline (misoprostol group) or 20 mL of normal saline only (saline group). The women were blinded to the group allocation, but the investigator who administered the injection was not. The trial was registered by the Research Ethics Committee of Hawler Medical University.

**Results:**

After umbilical vein injection, delivery of the placenta occurred in 91.3% of women in the misoprostol group and 69.5% of women in the saline group, which was not a significant difference between the two groups. The median vaginal blood loss from the time of injection until delivery of the placenta was significantly less in the misoprostol group (100 mL) than in the saline group (210 mL) (*p* value < 0.001).

**Conclusion:**

Umbilical vein injection of misoprostol is an effective treatment for retained placenta, and reduces the volume of vaginal blood loss with few adverse effects.

**Clinical Trial Registration:**

Current Controlled Trial HMU: N252.1.2011

## Background

### Definitions and maternal complications of retained placenta

No consensus exists regarding the normal length of the third stage of labour, or the time at which the placenta should be termed “retained” and intervention should be started [1].

The intrapartum guidelines published by the National Institute for Health and Clinical Excellence suggest intervention when the placenta has not been delivered within 30 minutes after birth with active management of the third stage of labour, or within 60 minutes after birth with physiological management of the third stage of labour [2].

Complications of RP include shock, postpartum haemorrhage, puerperal sepsis, and uterine subinvolution; which may result in the patient requiring hysterectomy [3].

In 1993, Herman first demonstrated ultrasonographically that retroplacental myometrial contraction is necessary to produce the shearing forces to the interface between the placenta and the myometrium that result in detachment of the placenta [4].

The current standard of management for RP is manual removal of the placenta (MROP), which usually requires general or regional anaesthesia at a hospital. MROP is an invasive procedure that may lead to serious complications such as haemorrhage, infection, and genital tract trauma. A simple and safe treatment for RP that can be administered at the location of the birth and reduces the need for MROP could be of major benefit to women worldwide [5].

### Administration of uterotonic drugs via the umbilical vein

The World Health Organization recommends umbilical vein injection of a uterotonic drug as the first line of treatment for RP. However, this treatment is not routinely used, probably because of lack of a large randomized controlled trial, and uncertainties regarding optimal drug and dosage regimens [6]. A Cochrane collaboration review found that umbilical vein injection of oxytocin is not effective for the treatment of RP [7]. A double-blind, placebo-controlled trial including women in the UK, Uganda, and Pakistan reported that umbilical vein injection of oxytocin had no clinically significant effect on the need for MROP [8].

### Study justification

All women should have access to simple and non invasive treatment for RP, whether delivering in a well-equipped hospital or a low-resource setting. To our knowledge, this is the first study of the effectiveness of umbilical vein injection of misoprostol for the treatment of RP to be conducted at a large maternity hospital. The Maternity Teaching Hospital is the only public hospital in Erbil City that manages high-risk pregnancies and deliveries. Most local women who develop complications after giving birth at home are treated at this hospital. The results of preliminary published trials suggest that administration of prostaglandins such as misoprostol may result in delivery of the placenta and reduced volume of blood loss in women with RP. This study evaluated the effectiveness of umbilical vein injection of misoprostol dissolved in normal saline versus normal saline only for the treatment of RP.

### Aims

The aims of this study were: (1) to determine whether umbilical vein injection of misoprostol for the treatment of RP reduces the need for MROP, and (2) to compare the volume of vaginal blood loss between women with RP who received umbilical vein injection of misoprostol and those who received normal saline only.

### Hypothesis

The primary hypothesis was that umbilical vein injection of misoprostol in women with RP despite active management of labour reduces the need for MROP under general anaesthesia. The secondary hypothesis was that umbilical vein injection of misoprostol in women with RP reduces the volume of vaginal blood loss.

## Methods

### Design and setting

This was a hospital-based, placebo-controlled clinical trial conducted at the Maternity Teaching Hospital in Erbil City, Northern Iraq from April 2011 to February 2012, including data collection and entering, follow-up of women, data analysis, and writing of the manuscript.

The Maternity Teaching Hospital is the only public maternity hospital in Erbil. This hospital provides obstetric services including management of high-risk pregnancies, medical terminations of pregnancy, and management of high-risk deliveries including caesarean deliveries. The hospital serves the entire population of the Erbil governorate, and is equipped to cope with emergency procedures 24 hours a day. The Directorate of Health in Erbil City reported 22,387 deliveries at the hospital during 2008, with an institutional delivery rate of 53.6%.

### Inclusion and exclusion criteria

**Figure 1:**
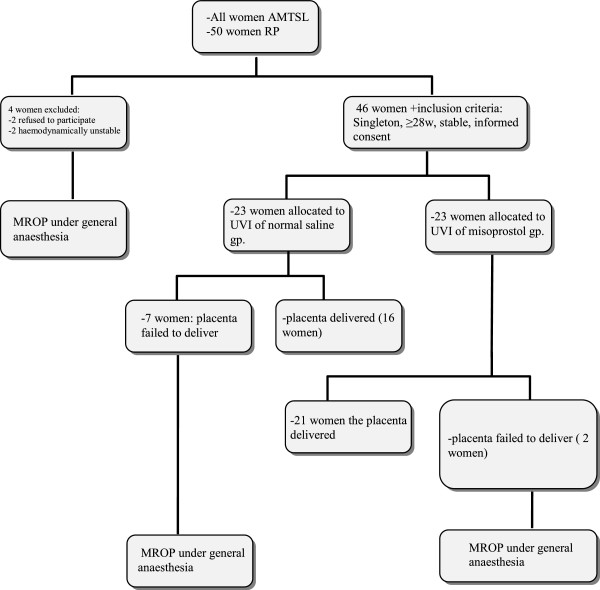
**Flowchart for trial entry.** Abbreviations: *AMTSL* active management of the third stage of labour, *RP* retained placenta, *MROP* manual removal of placenta, *UVI* umbilical vein injection.

All women with a singleton pregnancy who delivered vaginally after at least 28 weeks of gestation and had a prolonged third stage of labour (more than 30 minutes) despite active management (intramuscular administration of 5 IU oxytocin and controlled cord traction) were considered to have RP, and were eligible for inclusion in the study unless they had significant bleeding. The exclusion criteria were: multiple pregnancies, previous caesarean delivery, haemodynamic instability, severe anaemia (haemoglobin concentration <8 g/dL), chorioamnionitis, and refusal of consent for inclusion (Figure 1).

### Enrolment, sample size, and group allocation

Routine management at the Maternity Teaching Hospital includes active management of the third stage of labour. Eligible women were identified when the placenta was not delivered within 30 minutes after delivery of the infant.

Informed consent was obtained from all subjects after explanation of the study protocol. The study was approved and registered by the Research Ethics Committee and Scientific Committee of the College of Medicine, Hawler Medical University (N252.1.2011).

Two syringes were prepared every day: one containing misoprostol dissolved in 20 mL of normal saline and the other containing 20 mL of normal saline only.

Women were alternately allocated to receive umbilical vein injection of either misoprostol in normal saline (misoprostol group) or normal saline only (saline group). The women were blinded to the group allocation, but the investigator who administered the injection was not. Data were collected using a specially designed questionnaire, including the obstetric history, general examination findings, vital signs, obstetric and vaginal examination findings, and haemoglobin concentration.

### Umbilical vein injection technique

Umbilical vein injection was performed according to the Piping’s method, as follows. The cord was cut, and a size 10 paediatric nasogastric tube was advanced into the umbilical vein. If resistance was felt, the catheter was retracted 1–2 cm and then advanced further if possible. If the catheter could not be advanced further without using force, the injection was administered through the catheter at that time. If the majority of the catheter was inserted before resistance was felt, indicating that it had reached the placenta, the catheter was retracted 3–4 cm to ensure that the tip was in the umbilical vein and not in a placental vessel, before injection [9].

The prepared syringes contained either 800 mcg misoprostol (four Misotac® 200 mcg tablets) dissolved in 20 mL of normal saline or 20 mL of normal saline only. The cord was occluded by finger pressure around the catheter during injection, and was clamped with the catheter still in position after injection. If spontaneous delivery of the placenta did not occur, delivery by gentle cord traction was attempted at 15 and 30 minutes after injection. The time from umbilical vein injection to delivery of the placenta was recorded. Delivery of the placenta was assessed by clinical signs of placental separation and expulsion, as described by Rogers et al. [10]. The same procedures were followed in both treatment arms.

If placental delivery failed to occur within 30 minutes after the injection, or significant bleeding occurred, MROP was performed under general anaesthesia. The time and method of placental delivery (spontaneous by controlled cord traction or MROP) were recorded.

The volume of blood loss from the time of umbilical vein injection to delivery of the placenta was measured by placing a pad under the patient's buttocks. The pad weighed 45 g before use, and was weighed after delivery of the placenta using a dedicated electronic scale (up to 5 kg). The blood loss was recorded in mL (1 g =1 mL) [11].

Adverse effects after misoprostol administration such as shivering, fever, dizziness, vomiting, flushes, nausea, abdominal pain, and headache were recorded.

### Follow-up

All women in both treatment arms were followed up for 24 hours postpartum. Vital signs (blood pressure, pulse rate, temperature, and respiratory rate), uterine fundal height, abnormal vaginal bleeding, and abdominal pain were recorded. Tonics, analgesics, and antibiotics were prescribed before discharge if needed, according to the local hospital guidelines.

### Statistical analysis

All data were analysed using the Statistical Package for Social Sciences (SPSS), version 18. Proportions were compared between groups using the χ^2^ test. Mean values were compared using the student’s *T*-test. Mann–Whitney test was used to determine the median of blood loss vaginally. A *P* value of ≤ 0.05 was considered statistically significant.

## Results

RP is a relatively rare condition that may occur without any risk factors. Fifty women were diagnosed with RP during the study period, of which 46 met the criteria for inclusion in the study. Twenty-three women were allocated to each of the misoprostol and saline groups. In one woman in the misoprostol group, the cord detached from the placenta after injection.

### Patient characteristics

There were no significant differences between the misoprostol and saline groups in terms of mean age, parity, or gestational age. There were also no significant differences between the two groups in terms of risk factors for RP including history of dilatation and curettage, preterm labour, and history of RP (Table 1).

**Table 1:** Demographic characteristics and risk factors for retained placenta

**Characters and risk factors**	**Groups(*n*** **= 23, in each group)**	**Mean ± SD**	***P***
Age (years)	Mesoprostol group	24.52 ± 5.169	*.374
	Normal saline group	25.83 ± 4.668	
Parity	Mesoprostol group	1.30 ± 1.222	*.37
	Normal saline group	2.00 ± 0.953	
G. age (weeks)	Mesoprostol group	37.22 ± 2.876	*.559
	Normal saline group	37.61 ± 1.373	
History of dilatation and curettage	Mesoprostol group	4(17.4%)	**1.000
	Normal saline group	5(21.7%)	
History of preterm labour	Mesoprostol group	4(17.4%)	**0.347
	Normal saline group	1(4.3%)	
History of retained placenta	Mesoprostol group	1(4.3%)	**1.00
	Normal saline group	0(0%)	

*T-Test, **χ^2^ test.

### Outcomes after umbilical vein injection

After umbilical vein injection, the placenta was delivered in 21 of the 23 women in the misoprostol group, and in 16 of the 23 women in the saline group, which was not a significant difference between the two groups (Table 2).

**Table 2:** Comparisons of outcomes between the misoprostol and saline groups

**P value**	**Normal Saline group(*n*** **= 23)**	**Misoprostol group(*n*** **= 23)**	**Outcome**
*0.135	7(30.4)	2(8.7%)	Need for manual removal of placenta
<0.001+	210	100	Median of blood loss vaginally
	100-500	30-75	Range of blood loss vaginally (ml)
	7-30	5-23	Range time for placental separation after CCT(mint)

CCT: controlled cord traction.

*Fisher’s exact test, +Mann–Whitney test.

The median volume of blood loss from the time of umbilical vein injection until delivery of the placenta was significantly less in the misoprostol group (100 mL) than in the saline group (210 mL) using Mann–Whitney test.The range of vaginal blood loss vaginally was 30-75 ml while the rang time to delivery using controlled cord traction was (5-23 minutes) in the misoprostol group versus (7-30 minutes) in the normal saline group (Table 2).

The mean time from injection until delivery of the placenta was 12.61 ± 6.479 minutes in the misoprostol group and 13.17 ± 10.152 minutes in the saline group (*P =* 0.823).

### Adverse effects after umbilical vein injection of misoprostol

One of the 23 women who received misoprostol developed shivering that was attributed to misoprostol injection.

## Discussion

### Delivery of the placenta

The placenta was delivered after umbilical vein injection in 91.3% of the women who received misoprostol. The Cochrane review of umbilical vein injection for the treatment of RP found that injection of a prostaglandin solution resulted in a significant reduction in the rate of MROP compared with injection of saline solution, but did not result in differences in the volume of vaginal blood loss or adverse effects. However, the review was based on only two small trials including a total of 51 women, and both trials had low methodological quality [7].

Misoprostol is an analogue of prostaglandin E1 that interacts with specific receptors on myometrial cells, initiating a cascade of events including a change in calcium concentration that initiates myometrial contraction, and softening of the cervix, leading to expulsion of the uterine contents [12].

Umbilical vein injection of misoprostol may result in local action of misoprostol at the base of the placenta. The results of this study are comparable with those of a previous study that concluded that misoprostol was an effective treatment for RP compared with normal saline [4]. However, there was no significant difference in the rate of delivery of the placenta between the misoprostol and saline groups in this study, which may be due to the small sample size.

### Volume of blood loss

There was a significant difference in the median volume of vaginal blood loss after umbilical vein injection between the misoprostol group and the saline group. This finding differed from those of Bider et al. [13], who reported a mean volume of blood loss of 210 mL in the prostaglandin group and 231 mL in the saline group (p = 0.7). This difference may be due to the smaller sample size of their study (n = 17) and the different uterotonic drug used (a prostaglandin F2 alpha analogue).

### Time to delivery of the placenta

The mean time from umbilical vein injection until separation of the placenta was shorter in the misoprostol group (13.63 ± 5.29 minutes) than in the saline group (18.93 ± 5.89 minutes), but this was not a significant difference between the two groups. Rogers et al. reported no significant difference in the time to delivery of the placenta between the misoprostol and normal saline groups [14].

Harara et al. reported that the time to delivery of the placenta was significantly shorter in the misoprostol group (7.0 ± 2.2 minutes). This may be because they compared misoprostol with two other uterotonic drugs (ergometrine and oxytocin) rather than with normal saline as in the current study [8].

### Adverse effects of misoprostol

Only 1 of the 23 women who received misoprostol developed shivering. Bider et al. reported that 1 of 10 women experienced adverse effects (fever and shivering) after administration of a prostaglandin F2 alpha analogue [14].

### Limitations

Although this study had significant findings in terms of delivery of the placenta after umbilical vein injection, it had some limitations. The sample size was small because RP is a relatively rare obstetric complication. Larger trials conducted over longer periods of time will be required to obtain definitive results. This study was also prone to assessment bias, because the investigator who administered the umbilical vein injection was aware of the group allocation. However, the women were alternately allocated to the two groups, and were not previously known to the investigators.

## Conclusion

Umbilical vein injection of misoprostol dissolved in normal saline resulted in a reduced rate of MROP under general anaesthesia compared with injection of normal saline only.

## Competing interests

The authors declare that they have no competing interests.

## Authors’ contributions

SA contributed to the development of the trial protocol. SR drafted the manuscript and performed umbilical vein injection in all subjects. Both authors contributed to data analysis, interpretation of the results, and revisions of the manuscript, and approved the final manuscript.

## Pre-publication history

The pre-publication history for this paper can be accessed here:

http://www.biomedcentral.com/1471-2393/14/37/prepub
